# Development of an Injection Mold with High Energy Efficiency of Vulcanization for Liquid Silicone Rubber Injection Molding of the Fisheye Optical Lens

**DOI:** 10.3390/polym15132869

**Published:** 2023-06-29

**Authors:** Chil-Chyuan Kuo, Qing-Zhou Tasi, Song-Hua Hunag, Shih-Feng Tseng

**Affiliations:** 1 Department of Mechanical Engineering, Ming Chi University of Technology, No. 84, Gungjuan Road, New Taipei City 24301, Taiwan; 2 Research Center for Intelligent Medical Devices, Ming Chi University of Technology, No. 84, Gungjuan Road, New Taipei City 24301, Taiwan; 3 Department of Mechanical Engineering, Chang Gung University, No. 259, Wenhua 1st Rd., Guishan Dist., Taoyuan City 33323, Taiwan; 4 Li-Yin Technology Co., Ltd., No. 37, Lane 151, Section 1, Zhongxing Road, Wugu District, New Taipei City 24301, Taiwan; 5 Department of Mechanical Engineering, National Taipei University of Technology, No. 1, Sec. 3, Zhongxiao E. Rd., Da’an Dist., Taipei City 106344, Taiwan

**Keywords:** liquid silicone rubber, conformal heating channel, conformal cooling channel, curing rate, energy efficiency, sustainable manufacturing

## Abstract

Liquid silicone rubber (LSR) techniques are experiencing exponential growth, particularly in the field of high technology due to the low-temperature flexibility, superior heat stability, chemical resistance, and aging resistance of LSR components. Enhancing the curing rate of LSR parts in liquid silicone rubber injection molding is an important research topic. In this study, an injection mold with high energy efficiency of vulcanization for the liquid silicone rubber injection molding of a fisheye lens was developed and implemented. The LSR injection mold has a conformal heating channel (CHC) and conformal cooling channel (CCC) simultaneously. The function of CHC is to enhance the curing rate of a fisheye lens in the LSR injection molding to meet the requirements of sustainable manufacturing. The curing rates of a fisheye lens were numerically examined using the Moldex3D molding simulation software. It was found that the curing rate of the fisheye optical lens cured by injection mold with CHC was better than that of the injection mold with a conventional heating channel. The curing efficiency could be increased by about 19.12% when the heating oil temperature of 180 °C was used to cure the fisheye optical lens. The simulation results showed that the equation y = −0.0026x^3^ + 1.3483x^2^ − 232.11x + 13,770 was the most suitable equation for predicting the curing time (y) through the heating oil temperature (x). It was found that the trend of the experimental results was consistent with the simulation results. In addition, the equation y = −0.0656x^2^ + 1.5827x − 0.894 with the correlation coefficient of 0.9974 was the most suitable equation for predicting the volumetric shrinkage of the fisheye optical lens (y) through the heating oil temperature (x). The volume shrinkage of the fisheye optical lens cured by injection mold with CHC was very similar to that of the injection mold with a conventional heating channel. The maximum volume shrinkage of the fisheye optical lens cured at 180 °C was about 8.5%.

## 1. Introduction

A liquid silicone rubber (LSR) is a two-component platinum-cured silicone, ideally formulated for the production of parts in technical demands, such as medical, aerospace, automotive, electrical, and consumer industries through liquid injection molding (LIM) [1,2]. LIM is a cost-effective approach to the mass production of LSR components or parts with complex geometries due to its ease of flowability [3]. Liquid silicone rubber injection molding [4] requires special treatment because of the thermosetting nature of the LSR material [5], such as intensive distributive mixing, before it is injected into the heated mold cavities and vulcanized. Conformal cooling channels (CCC) are widely used in injection molding to improve productivity [6,7,8]. In practice, a major disadvantage is that the curing rate of LSR parts is limited due to conventional LSR injection molds being incorporated with cartridge heaters [9]. In practice, the curing rate of the molded part in LIM is a major concern, especially for thick-walled LSR parts. To enhance the curing rate of a fisheye lens in LIM, an innovative conformal heating channel (CHC) was proposed in this study. Metal additive manufacturing (AM) [10,11] techniques can create LSR injection molds with CHC and CCC. However, there are many distinct processing defects, including cracking [12], warpage [13], and residual stress [14]. Additionally, little has been reported on the injection mold with CCC and CHC simultaneously hitherto.

In this study, two sets of LSR injection molds were designed. The curing rates of a fisheye lens were then numerically examined using the Moldex3D molding simulation software. To validate the simulation results, two sets of LSR injection molds were fabricated using rapid tooling technology [15,16,17,18]. During the curing phase of a fisheye lens, an infrared thermal imager was used to record temperature history during LIM.

## 2. Experimental Details

**Figure 1:**
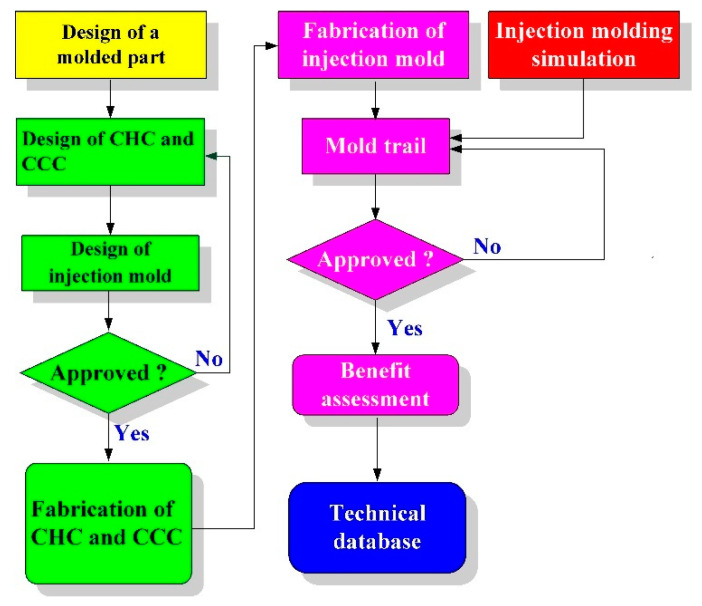
Flowchart of this study.

**Figure 2:**
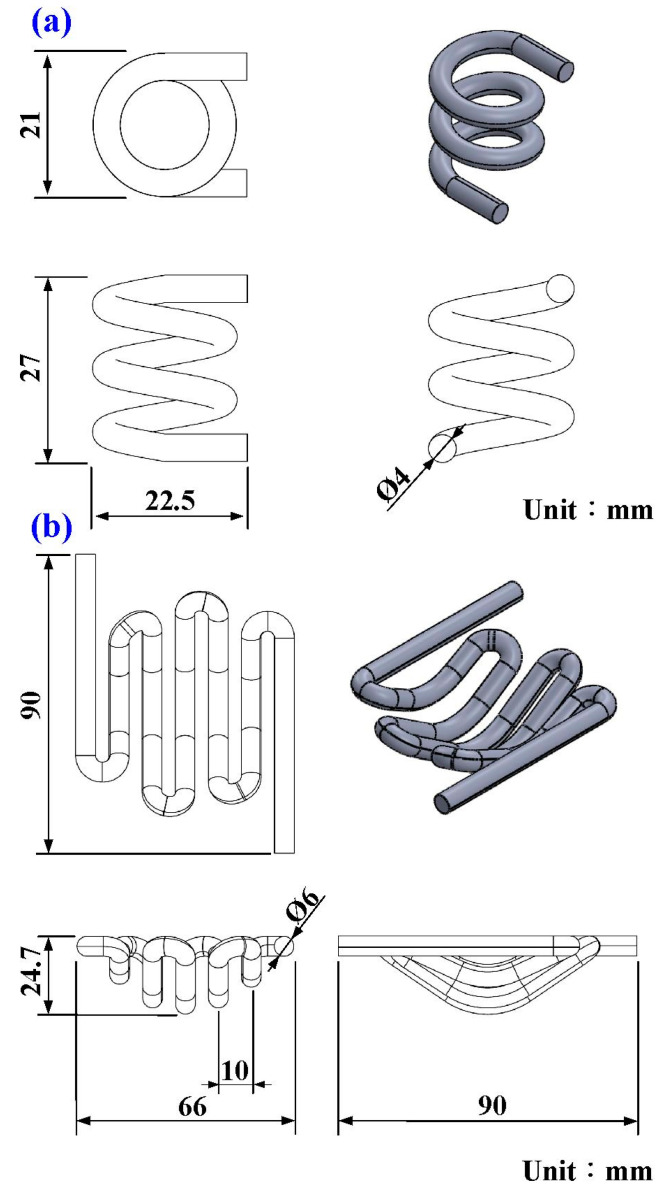
3D CAD model and dimensions of (**a**) CCC and (**b**) CHC.

**Figure 3:**
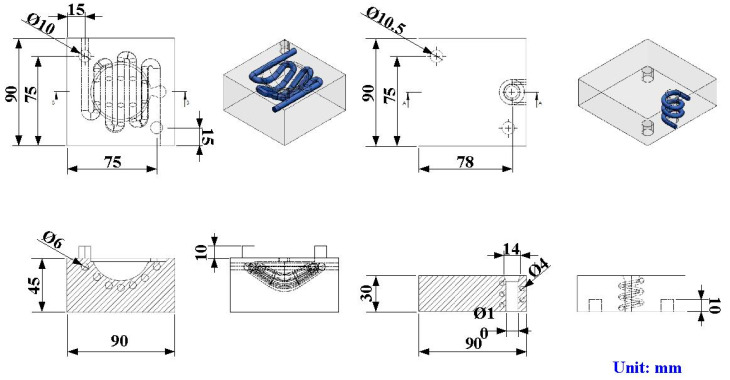
CAD models of cavity insert with CHC and core insert with CCC.

A fisheye lens was used as the molded part in this study. The molded part, LSR injection molds, CHC, and CCC were designed using the three-dimensional (3D) modeling software (Parametric Technology Corporation, Boston, MA, USA). Figure 1 shows the flowchart of this study. The diameter of the molded part was around 50 mm and the thickness of the center was around 18.7 mm. Figure 2 shows the 3D CAD model and dimensions of CCC and CHC. Figure 3 shows the CAD models of cavity insert with CHC and core insert with CCC. The core insert of the LSR injection mold had a length of 90 mm, a height of 30 mm, and a width of 90 mm. The cavity insert of the LSR injection mold had a length of 90 mm, a height of 45 mm, and a width of 90 mm. The cross-section of CCC and CHC was circular. The diameter of CCC and CHC was about 4 mm and 6 mm, respectively. The distance between the wall of CHC to the mold surface was about 8 mm. The pitch distance between central lines of CHC was about 10 mm. The distance between the wall of CCC to the mold surface was about 8 mm. The main purpose of the CCC was to maintain the molding material of LSR in a liquid state in the filling system. Two sets of LSR injection molds were fabricated by aluminum-filled epoxy resin (TE-375, Jasdi Inc., New Taipei City, Taiwan). In one, the LSR injection mold possessed a CHC and CCC. In the other, the LSR injection mold had conventional heating channels and CCC.

**Figure 4:**
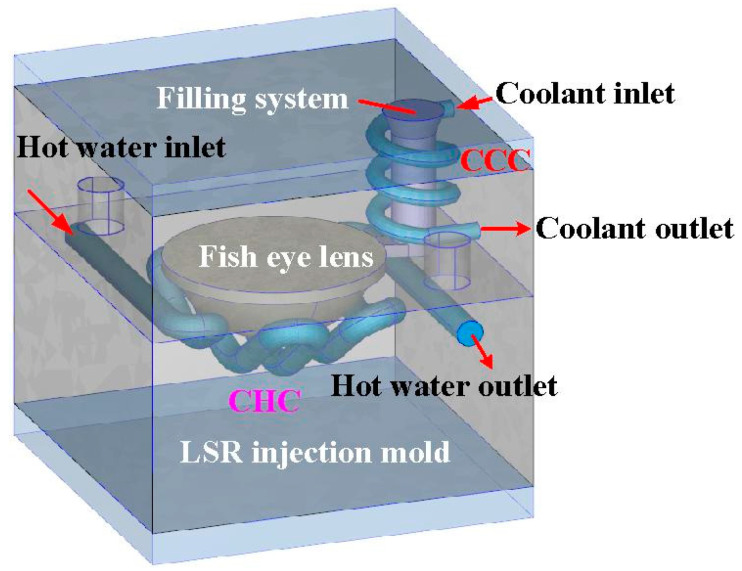
Schematic illustration of LSR injection mold with CHC and CCC for LIM.

**Figure 5:**
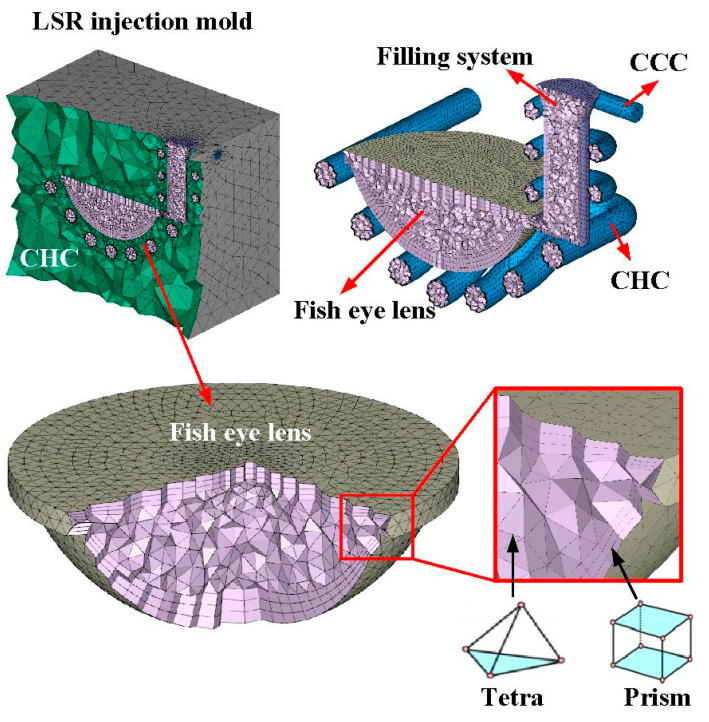
Mesh of the simulation models used in this study.

**Figure 6:**
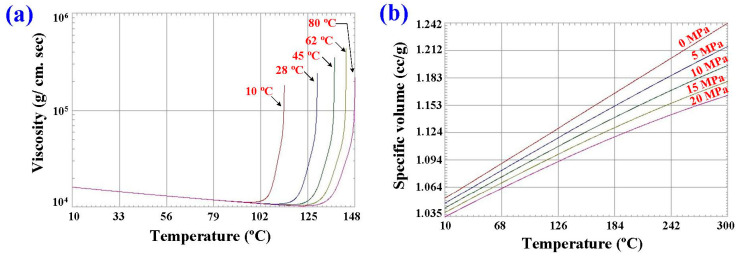
Material properties of LSR used in this study: (**a**) viscosity chart and (**b**) pressure–volume–temperature diagram.

**Figure 7:**
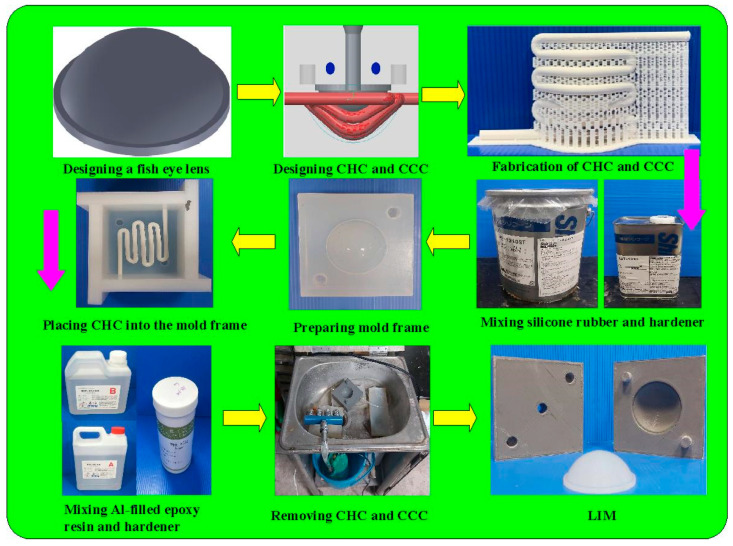
Manufacturing processes of LSR injection mold.

**Figure 8:**
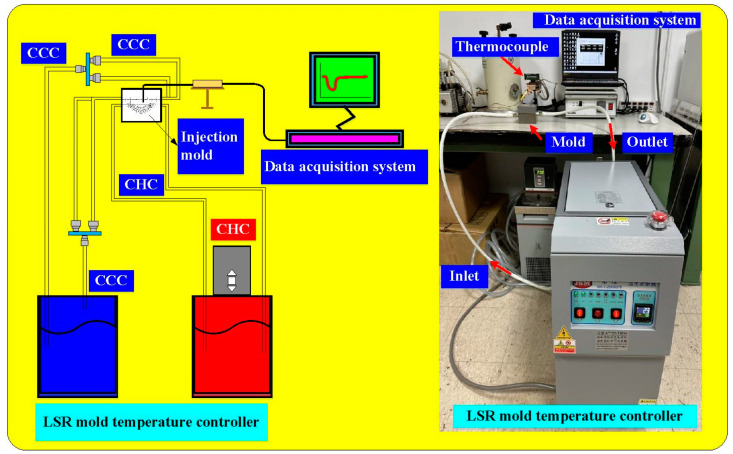
Experimental setup for LSR injection molding.

In this study, the Moldex3D simulation software (R14 SP3OR, CoreTech System Inc., New Taipei City, Taiwan) was used to investigate the curing rate of the fisheye lens. Figure 4 shows the schematic illustration of LSR injection mold with CHC and CCC for LIM. Figure 5 shows the mesh of the simulation models used in this study. The numbers of meshes for the LSR injection mold, fisheye optical lens, filling system, and both channels were 1,344,551, 2,216,121, 5800, and 477,176, respectively. Figure 6 shows the material properties of the LSR used in this study. The viscosity depends on temperature, shear rate, and pressure, since it is the index of the resistance of an LSR to flow. Generally, the LSR undergoes a significant volumetric change over temperature and pressure. Characterization of the pressure–volume–temperature relationship is required to calculate warpage or shrinkage of a fisheye lens after solidification. Figure 7 shows the manufacturing processes of an LSR injection mold. Thanks to the features of additive manufacturing technology, both CCC and CHC were printed with polyvinyl butyral (PVB) filament [19,20] using a fused deposition modeling (FDM) machine [21,22] (Teklink smart solution Inc., New Taipei City, Taiwan). The printing parameters were a printing temperature of 200 °C, bed temperature of 60 °C, layer thickness of 0.1 mm, and printing speed of 30 mm/s. A bridge mold was firstly fabricated by silicone rubber (KE-1310ST, Shin Etsu Inc., New Taipei City, Taiwan) and hardener. The printing times of the conventional heating channel and CHC were approximately 82 and 196 min, respectively. The amount of printing materials used in the conventional heating channel and CHC were approximately 10 and 14 g. A computer program using Visual Basic was developed to reduce human error in determining the amounts of both base compound and hardener accurately. It should be noted that the LSR injection molds were fabricated by a developed mixture which was comprised of epoxy resins (EP-2N1, Ruixin Inc., New Taipei City, Taiwan) and 41 vol.% Al powder with an average particle size of about 45 µm. The mixture was then placed in the vacuum pump (F-600, Feiling Inc., New Taipei City, Taiwan) to eliminate air bubbles derived from the mixing process. Figure 8 shows the experimental setup for LSR injection molding. The thermoset material used in both the experiment and simulation was the ELASTOSIL^®^ LR 3003/50 US A/B (Wacker Chemical AG Inc., Munich, Germany). This system comprises CCC, CHC, a heating oil temperature controller (JSO—1020E, Jie-Seng Inc., New Taipei, Taiwan), three k-type thermocouples (C071009-079, Cheng Tay Inc., Taipei, Taiwan) with a measurement sensitivity of ±1 °C, a water reservoir with a thermoelectric cooler (TEC12706AJ, Caijia Inc., Taipei City, Taiwan), and a data acquisition system (MRD-8002L, IDEA System Inc., Taipei City, Taiwan). The upper limit of the heating oil temperature in the mold temperature controller was 180 °C. To investigate the effect of heating oil temperature on the curing rate of a fisheye lens, nine different heating oil temperatures, i.e., 60, 80, 100, 120, 140, 150, 160, 170, and 180 °C, were used in this study. The volume flow rate was approximately 120–130 cc/s. The coolant temperature was about 26–28 °C. Note that the surface temperature of the fisheye lens was monitored by an infrared camera (BI-TM-F01P, Panrico Trading Inc., New Taipei City, Taiwan).

## 3. Results and Discussion

**Figure 9:**
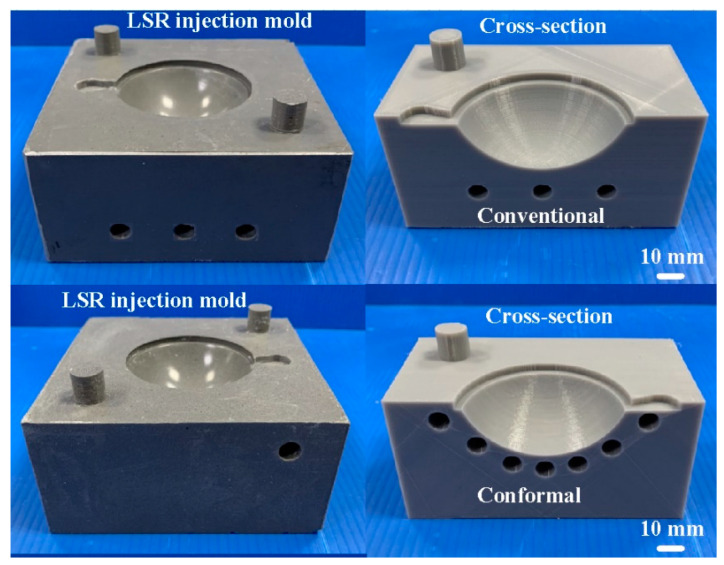
LSR injection mold.

**Figure 10:**
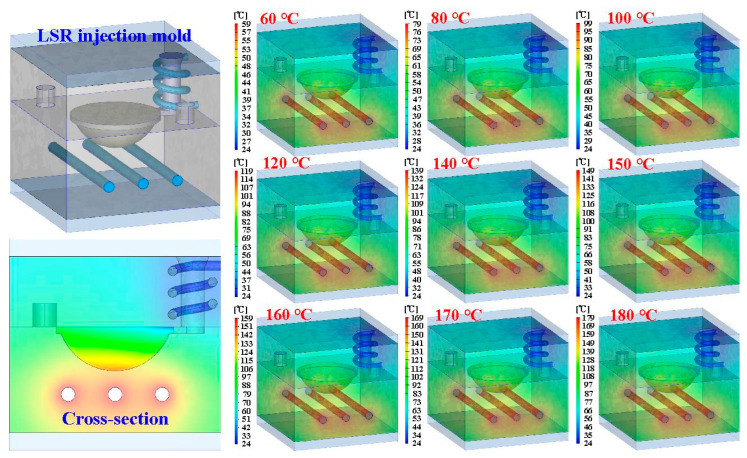
Mold temperature distribution of LSR injection mold with conventional heating channel using nine different heating oil temperatures.

**Figure 11:**
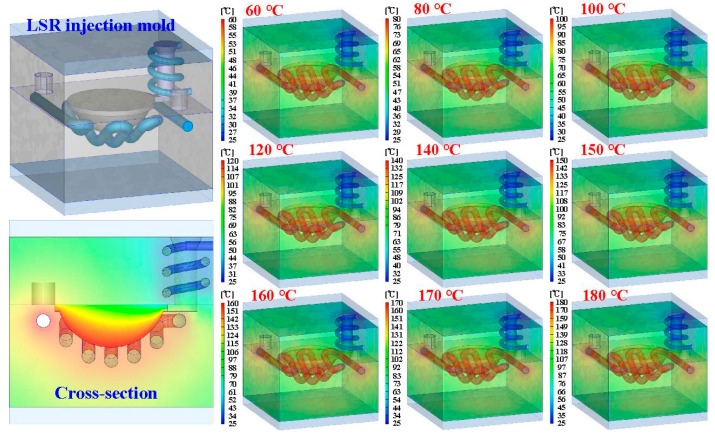
Mold temperature distribution of LSR injection mold with CHC using nine different heating oil temperatures.

**Figure 12:**
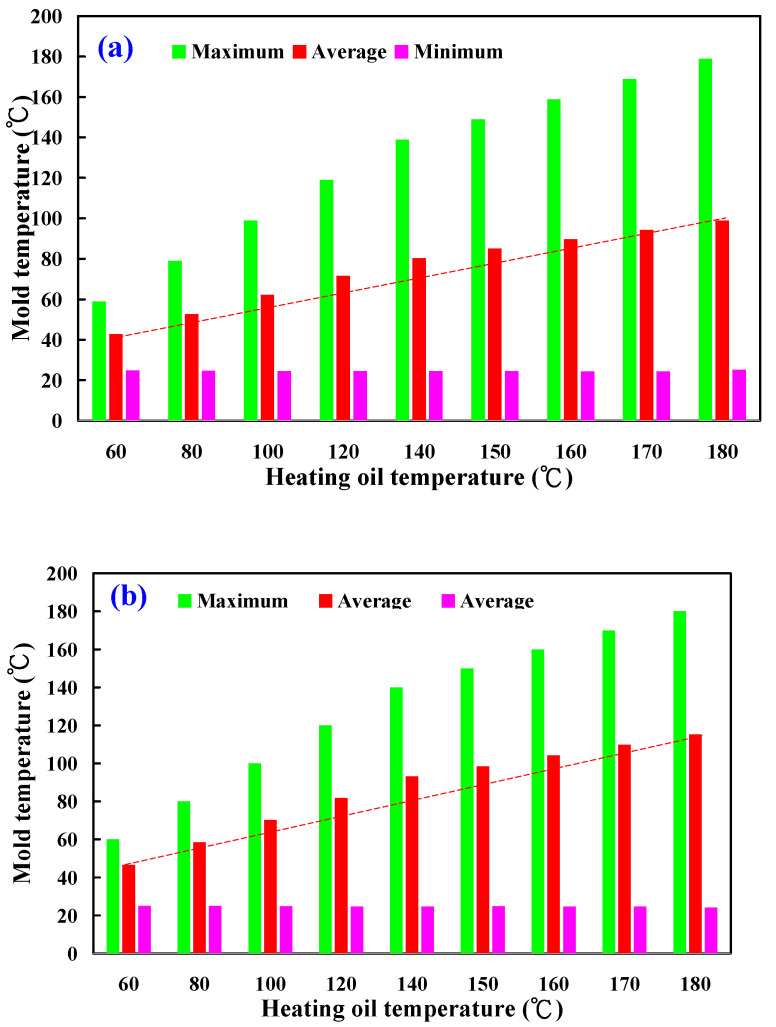
Dependence of the mold temperature on the different heating oil temperature for the LSR injection mold with (**a**) conventional heating channel and (**b**) CHC using nine different heating oil temperatures.

To see the inside of the LSR injection mold, the mold was cut in half using a bandsaw. Figure 9 shows the LSR injection mold. Figure 10 shows the mold temperature distribution of the LSR injection mold with a conventional heating channel using six different heating oil temperatures: 40, 50, 55, 60, 65, and 70 °C. The average mold temperature could reach about 43, 53, 63, 72, 81, 86, 89, 94, and 99 °C, respectively. Figure 11 shows the mold temperature distribution of the LSR injection mold with CHC using six different heating oil temperatures: 40, 50, 55, 60, 65, and 70 °C. The average mold temperature could reach about 47, 59, 71, 82, 94, 99, 105, 110, and 116 °C, respectively. Figure 12 shows the dependence of the mold temperature on the different heating oil temperatures for the LSR injection mold with a conventional heating channel and CHC. It can be seen that the average mold temperature for LSR injection mold with CHC could be increased by approximately 8.6%, 11%, 12.6%, 14.1%, 15.9%, 15.6%, 16%, 16.4%, and 16.5%, respectively. Based on these results, two phenomena were found. One is that the heat transfer effect on the mold surface for the LSR injection mold with CHC was significantly better than that of the mold surface heat transfer effect for the LSR injection mold with a conventional heating channel. It can be asserted that the fisheye optical lens curing effect of the LSR injection mold with CHC is obviously better than that of the LSR injection mold with conventional heating channel. The other is that the curing direction of the fisheye optical lens for the LSR injection mold with a conventional heating channel was solidified from the bottom of the fisheye optical lens to the top of the fisheye optical lens. However, the curing direction of the fisheye optical lens for the LSR injection mold with CHC was along the contour of the fisheye optical lens from outside to the inside.

**Figure 13:**
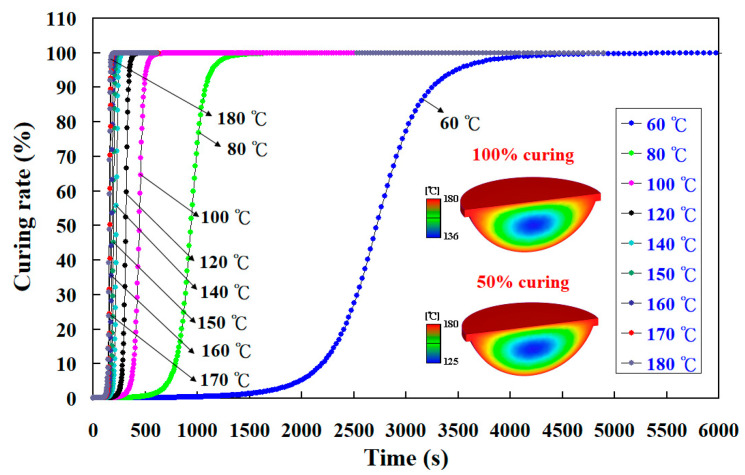
Numerical simulation results of the curing rate of the fisheye optical lens as a function of curing time for LSR injection mold with conventional heating channel using different heating oil temperatures.

**Figure 14:**
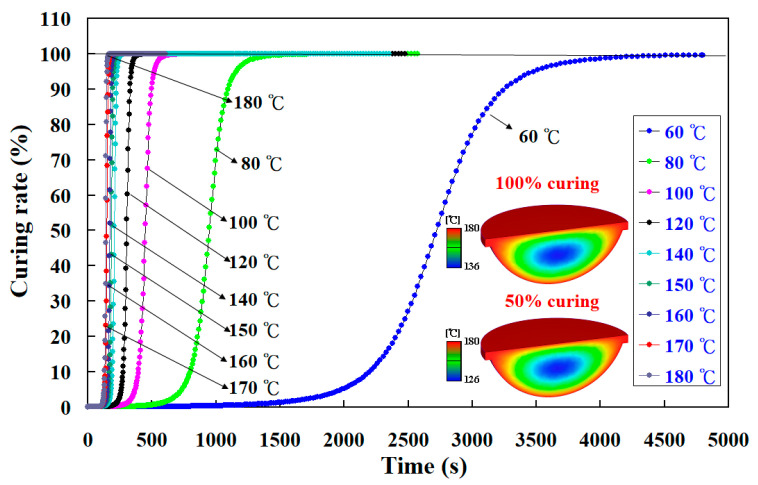
Numerical simulation results of the curing rate of the fisheye optical lens as a function of curing time for LSR injection mold with CHC using different heating oil temperatures.

To investigate the curing kinetics of the fisheye optical lens, nine different heating oil temperatures were performed in this study using a numerical simulation method. Figure 13 shows the numerical simulation results of the curing rate of the fisheye optical lens as a function of curing time for the LSR injection mold with a conventional heating channel using different heating oil temperatures. Figure 14 shows the numerical simulation results of the curing rate of the fisheye optical lens as a function of curing time for the LSR injection mold with CHC using different heating oil temperatures. The curing times required for the fisheye optical lens to reach 100% complete curing for LSR injection mold with conventional heating channel using heating oil temperatures of 60, 80, 100, 120, 140, 150, 160, 170, and 180 °C were about 5683 s, 2473 s, 2048 s, 626 s, 553 s, 446 s, 385 s, 329 s, and 319 s, respectively. The curing times required for the fisheye optical lens to reach 100% complete curing for the LSR injection mold with CHC using heating oil temperatures of 60, 80, 100, 120, 140, 150, 160, 170, and 180 °C were about 4161 s, 2308 s, 1723 s, 520 s, 464 s, 427 s, 369 s, 305 s, and 258 s, respectively. It was found that the time for the fisheye optical lens to reach 100% complete curing can be saved by approximately 27.78%, 6.678%, 15.878%, 16.938%, 16.098%, 4.268%, 4.168%, 7.298%, and 19.12%, respectively. This result revealed that the curing rate of the fisheye optical lens cured by injection mold with CHC is better than that of the injection mold with a conventional heating channel. The curing efficiency could be increased by about 19.12% when the heating oil temperature of 180 °C was used to cure the fisheye optical lens. 

**Figure 15:**
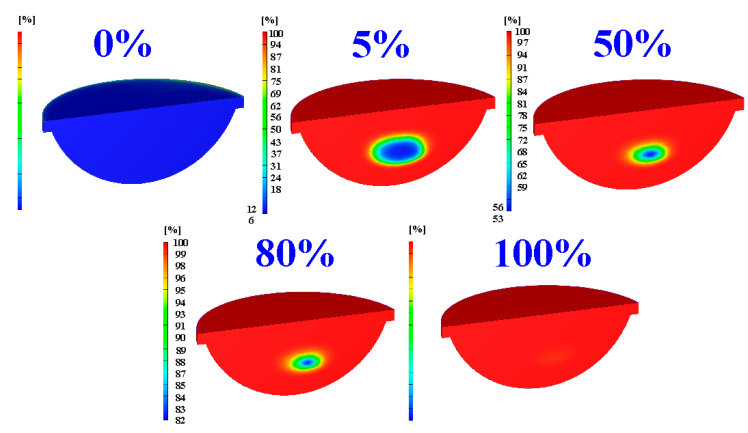
Numerical simulation results of the evolution of the curing rate of the fisheye optical lens using heating oil temperature of 180 °C.

**Figure 16:**
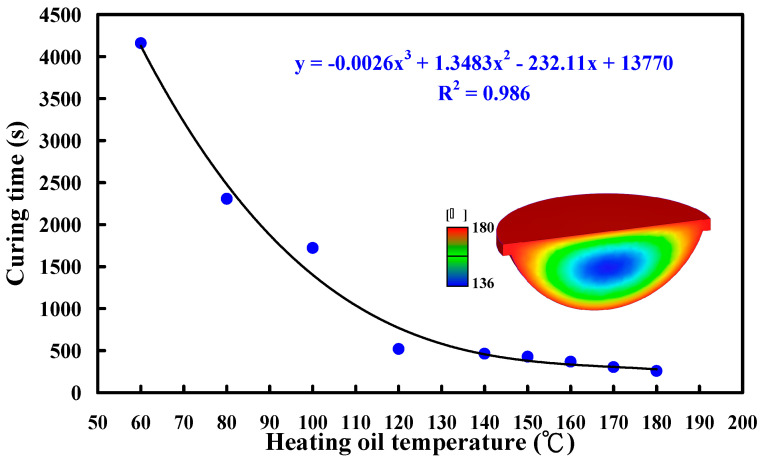
Dependence of the heating oil temperature and curing time of the fisheye optical lens.

Figure 15 shows the numerical simulation results of the evolution of the curing rate of the fisheye optical lens using a heating oil temperature of 180 °C. Figure 16 shows the dependence of the heating oil temperature and curing time of the fisheye optical lens. It should be noted that the equation y = −0.0026x^3^ + 1.3483x^2^ − 232.11x + 13,770 with the correlation coefficient (R^2^) of 0.986 was the most suitable equation for predicting the curing time (y) through the heating oil temperature (x).

**Figure 17:**
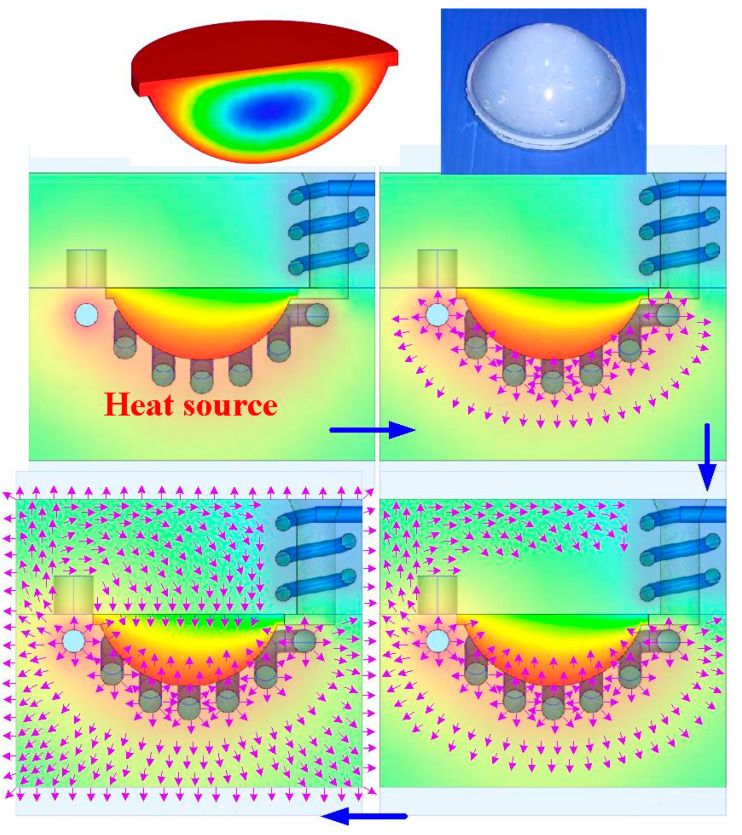
Schematic illustration of heat transfer of LSR injection mold with heating oil channel.

**Figure 18:**
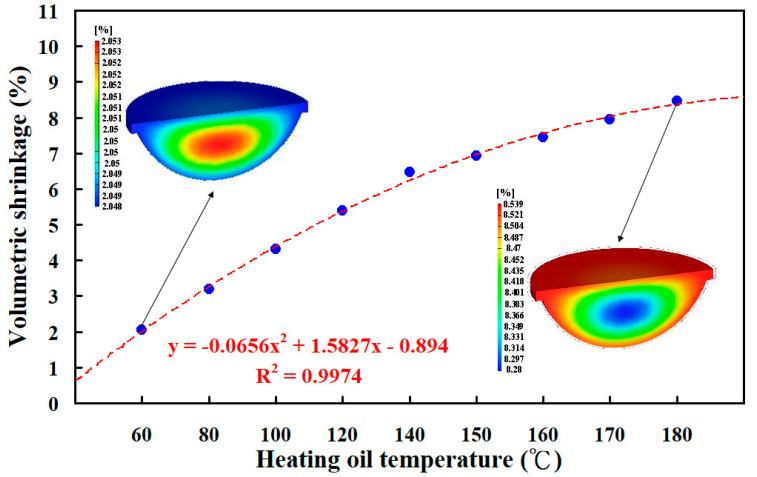
Numerical simulation results of volumetric shrinkage of the fisheye optical lens for the LSR injection mold with CHC using nine different heating oil temperatures.

According to the numerical simulation results, the schematic illustration of the heat transfer of LSR injection mold with the heating oil channel is shown in Figure 17. Note that the arrows indicate the heat transfer direction. The heat source of the LSR injection mold is the heating oil inside the CHC. The heat is not only transferred to the cavity of the LSR injection mold, but also to the LSR injection mold when the heating oil is passed into the CHC. It is well known that the thermal conductivity of the LSR injection mold is higher than that of LSR. The heat will be transferred to the core of the LSR injection mold, resulting in the top and bottom of the fisheye optical lens being cured simultaneously. The center of the fisheye optical lens will be cured last. Therefore, the solidification time is defined when the center of the fisheye optical lens is fully cured. Figure 18 shows the numerical simulation results of the volumetric shrinkage of the fisheye optical lens for the LSR injection mold with CHC using nine different heating oil temperatures. Two phenomena were found. One is that the volumetric shrinkage of the fisheye optical lens is significantly related to the temperature of the heating oil, since the higher the temperature of the heating oil, the higher the volumetric shrinkage of the fisheye optical lens. The other is that the volumetric shrinkage of the fisheye optical lens is not related to the configuration of the heating channels, since the volumetric shrinkage of the fisheye optical lens is very similar when both CHC and conventional heating cannels were employed to cure the fisheye optical lens. It was found that the equation y = −0.0656x^2^ + 1.5827x − 0.894 with the correlation coefficient of 0.9974 was the most suitable equation for predicting the volumetric shrinkage of the fisheye optical lens (y) through the heating oil temperature (x).

**Figure 19:**
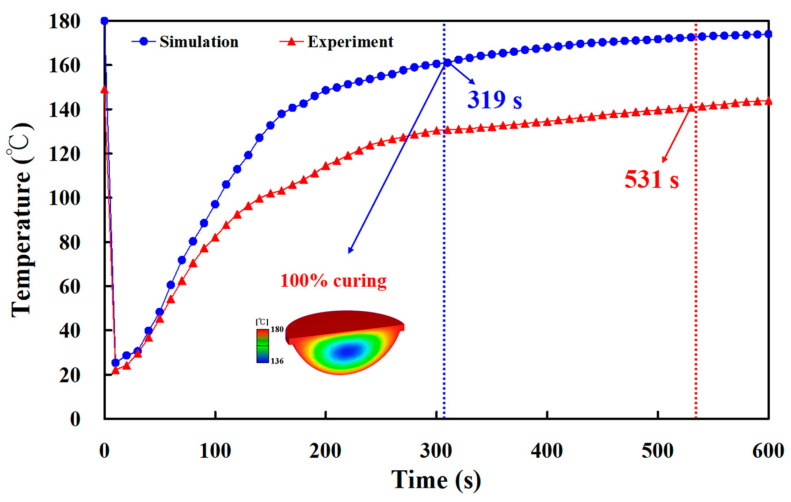
Experimental and numerical simulation results of curing time of fisheye lens for LSR injection mold with conventional heating channel.

**Figure 20:**
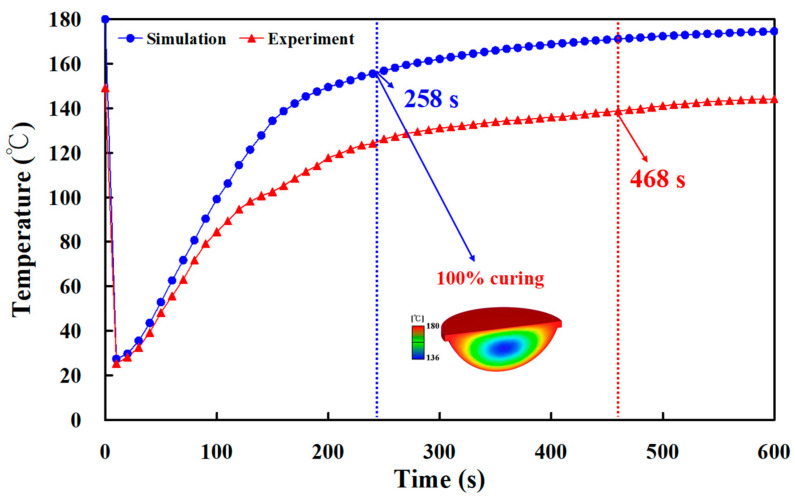
Experimental and numerical simulation results of curing time of fisheye lens for LSR injection mold with CHC.

**Figure 21:**
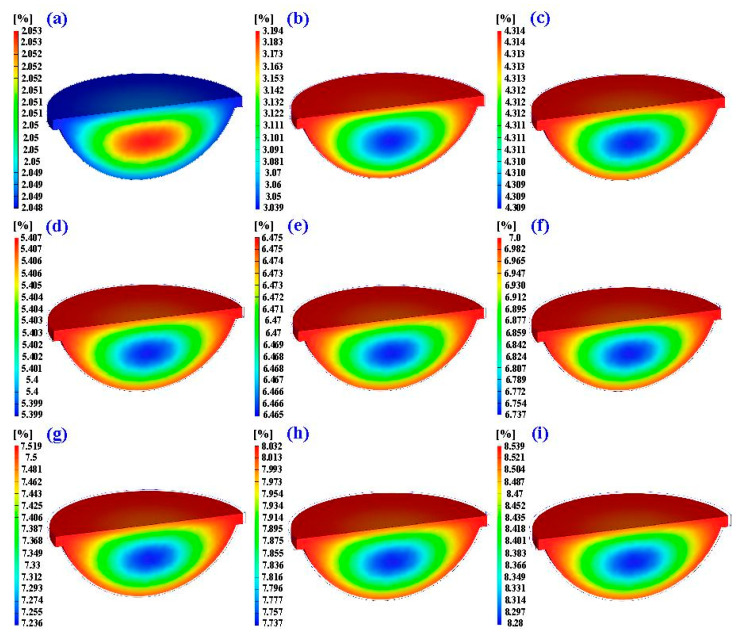
Numerical simulation results of the volume shrinkage of the fisheye optical lens for LSR injection mold with CHC under the curing temperature at (**a**) 60 °C, (**b**) 80 °C, (**c**) 100 °C, (**d**) 120 °C, (**e**) 140 °C, (**f**) 150 °C, (**g**) 160 °C, (**h**) 170 °C, and (**i**) 180 °C.

Figure 19 shows the experimental and numerical simulation results of the curing time of a fisheye lens for an LSR injection mold with conventional heating channel. It was found that the curing time of a fisheye lens obtained by simulation and experiment was 319 s and 531 s, respectively. The simulation error rate was only about 39%. Figure 20 shows the experimental and numerical simulation results of the curing time of a fisheye lens of the LSR injection mold with CHC. Indeed, only two experiments were carried out since LSR injection molds cannot withstand long-term high-temperature experiments. It was found that the curing times of a fisheye lens obtained by simulation and experiment were 258 s and 468 s, respectively. As a result, the trend of the experimental results is consistent with the simulation results. However, the simulation error rate was about 44% due to the difference in both boundary and initial conditions between experiment and simulation. To reduce the simulation error rate, future research will focus on setting both the boundary and initial conditions in the simulation software. Figure 21 shows the numerical simulation results of the volume shrinkage of the fisheye optical lens of the LSR injection mold with CHC and conventional heating channels. The maximum volume shrinkage of the fisheye optical lens cured at 60, 80, 100, 120, 140, 150, 160, 170, and 180 °C was about 2.1%, 3.2%, 4.3%, 5.4%, 6.5%, 7%, 7.5%, 8%, and 8.5%, respectively. Two phenomena were found. One is that the volume shrinkage of the fisheye optical lens is related to the heating oil temperature. The higher the heating oil temperature, the greater the volume shrinkage of the fisheye optical lens. The other is that the volume shrinkage of the fisheye optical lens is not related to the layout of the heating channel because the volume shrinkage of the fisheye optical lens of the LSR injection mold with CHC and conventional heating channels is very similar.

In this study, an injection mold with high energy efficiency of vulcanization for the LSR injection molding of a fisheye optical lens has been designed, fabricated, and evaluated. It can be concluded that the developed LSR injection mold meets the goals of sustainable development [23] (SDG_S_ 7, 8, and 12) [24,25,26,27,28]. Thus, the findings provide the greatest application potential in the research and design stage of an LSR injection mold. In this study, the LSR injection mold was fabricated with a mixture comprised of epoxy resins composites filled with Al powder. However, the mechanical properties [29] of the LSR injection mold were lower than those of the LSR injection mold fabricated with conventional tool steel [30]. Therefore, improving the mechanical properties of an LSR injection mold by adding some distinctive reinforcing filler [31,32,33,34,35] in the mixture is an important research direction for future study. To elucidate these points, further work must be done. The fabricated fisheye optical lens was lighter compared with an optical lens fabricated from glass. It should be pointed out that the fabricated fisheye optical lenses can be used to replace glass lenses [36]. These research topics are currently being investigated and the results will be presented in a later work.

## 4. Conclusions 

LSR is widely applied in manufacturing optical components, automotive parts, medical devices, and household goods. In this study, LSR injection molds with high energy efficiency of vulcanization were fabricated through the integration of rapid tooling and additive manufacturing technologies. The curing rates were numerically examined using the Moldex3D molding simulation software. An infrared thermal imager was used to record temperature history during curing of a fisheye lens. The main conclusions from the experimental work in this study are as follows:

1. A LSR injection mold with high energy efficiency of vulcanization can provide potential applications in the LSR injection molding industry because it meets the requirement of sustainable manufacturing.
2. The curing efficiency can be increased by about 19.12% when the heating oil temperature of 180 °C is used to cure the fisheye optical lens.
3. The equation y = −0.0026x^3^ + 1.3483x^2^ − 232.11x + 13,770 is the most suitable equation for predicting the curing time (y) through the heating oil temperature (x). The trend of the experimental results is in good agreement with the simulation results.
4. The equation y = −0.0656x^2^ + 1.5827x − 0.894 with the correlation coefficient of 0.9974 is the most suitable equation for predicting the volumetric shrinkage of the fisheye optical lens (y) through the heating oil temperature (x). The volume shrinkage of the fisheye optical lens cured by the injection mold with CHC is very similar to that of the injection mold with a conventional heating channel.

## Data Availability

Data and materials are available.
